# Purrble Use Among LGBTQ+ Young People With Self-Harm Experiences: Findings From a Qualitative Process Evaluation of a Randomized Controlled Trial

**DOI:** 10.2196/103590

**Published:** 2026-10-27

**Authors:** A Jess Williams, Ellen Townsend, Lauren Posada, Seonaid Cleare, Lucy Martin, Amelia Chapman-Nisar, Chris Hollis, James J Gross, Petr Slovak

**Affiliations:** 1 National Centre for Suicide Prevention and Self-Harm Research, Population Data Science, School of Medicine, Swansea University, Singleton Campus, Swansea, Wales, SA2 8PP, United Kingdom, 44 7969432592; 2 Department of Informatics, King's College London, London, England, United Kingdom; 3 Institute of Mental Health, University of Nottingham, Nottingham, England, United Kingdom; 4 School of Psychology, University of Nottingham, Nottingham, England, United Kingdom; 5 University of Glasgow, Glasgow, Scotland, United Kingdom; 6 National Institute of Health Research (NIHR) MindTech MedTech Research Centre, School of Medicine, University of Nottingham, Nottingham, England, United Kingdom; 7 Stanford University, Stanford, CA, United States; 8 Department of Psychiatry, University of Oxford, Oxford, England, United Kingdom

**Keywords:** process evaluation, socially assistive robot, qualitative, self-harm, LGBTQ+, young people, lesbian, gay, bisexual, transgender, queer, or questioning

## Abstract

**Background:**

Difficulties with emotion regulation (ER) have widely been associated with the development and persistence of poor mental health, such as depression, anxiety, self-harmful thoughts, behaviors, and suicide attempts. Despite the importance of ER as a transdiagnostic risk factor, few interventions directly target ER. The impact of one such intervention, Purrble (a socially assistive robot; developed by Sproutel and Committee for Children), was recently evaluated when compared to treatment-as-usual (TAU) within a randomized controlled trial (RCT) with sexual orientation and gender identity minorities, that is, lesbian, gay, bisexual, transgender, queer, or questioning (LGBTQ+) youth, who had self-harm experiences. However, while RCTs are well suited to evaluating effectiveness, they offer limited insight into how participants engage with, integrate, and perceive the value of interventions, like Purrble, in their everyday lives. Consequently, complementary qualitative process evaluation is needed to fully understand participants’ experiences and contextualize trial outcomes.

**Objective:**

This study aimed to explore and understand the engagement with and integration of the Purrble intervention using process evaluation interviews, considering its perceived influence on ER, mental health, and self-harm experiences.

**Methods:**

Following their participation in the Purrble RCT, 40 LGBTQ+ young people (16‐25 years) with current self-harm ideation took part in this qualitative process evaluation using semistructured online interviews. Interviews explored participant expectations of the RCT and Purrble; engagement and appropriation of Purrble; and perceived impact on mental health and self-harm. Thematic analysis was used to develop a thematic framework.

**Results:**

Four overarching themes were developed from the data. The first theme describes how participants used Purrble to ground themselves and manage distress, with participants suggesting de-escalation of their self-harm thoughts or behaviors. The second theme captures how Purrble was perceived as a companion or source of comfort, supporting self-soothing and self-care. The third theme highlights the role of accessibility and proximity, how the availability and location of Purrble during moments of distress influenced its perceived usefulness. The final theme examines circumstances in which participants chose not to engage with Purrble, offering insights into barriers to use and individual differences in engagement.

**Conclusions:**

This process evaluation helps to contextualize how Purrble could be a helpful intervention for those LGBTQ+ young people with experience of self-harm thoughts and behaviors. This evaluation also clarifies when Purrble may not be helpful. These qualitative findings offer important contextual insights alongside the quantitative results of the related RCT.

## Introduction

Lesbian, gay, bisexual, transgender, queer, or questioning (LGBTQ+) young people are at increased risk for poor mental health outcomes, including elevated rates of depression, anxiety, self-harm, and suicide [1-6]. In the United Kingdom alone, an estimated 65.3% of LGBTQ+ youth have a history of self-harm [2]. Internationally, LGBTQ+ young people are also at increased risk of suicide [7], being up to 6 times more likely to attempt suicide than their cisgender, heterosexual peers [1,8].

Despite elevated risk, help-seeking among LGBTQ+ youth remains low [3], often due to fears of disclosing self-harm, confidentiality concerns, prior discrimination, and negative or uninformed provider responses [9-12]. These individual and systemic barriers highlight the potential value of digital interventions, to provide accessible, anonymous, and stigma-free support [13,14]. Digital interventions, delivered via computers, smartphones, wearable devices, or socially assistive technologies, offer a means of accessing support without direct interpersonal contact [15], reducing exposure to discrimination and encouraging anonymized help-seeking [14].

Purrble, a socially assistive robot developed by Sproutel and Committee for Children, was co-designed with children to support emotion regulation (ER), represents 1 such digital intervention. Difficulties in ER are a well-known transdiagnostic risk factor for poor mental health and self-harm [16-19]. Purrble supports in-situ regulation of emotions immediately through intuitive interaction requiring no explicit instruction or guidance [20,21]. In short, Purrble simulates a heartbeat which responds to touch, changing from frantic vibrations accompanied by squeaking noises to gentle “purring” as it is “soothed” by the user’s gentle touch.

Early evidence suggests Purrble may help young people manage distress by facilitating real-time ER strategies [22-24]. A recent feasibility study with LGBTQ+ youth found that Purrble may help reduce distress and potentially prevent self-harm [22]. However, given the small sample size, further exploration was warranted.

To address this need, a randomized controlled trial (RCT) was conducted exploring the impact of a socially assistive robot, Purrble, with young LGBTQ+ people who had thoughts of self-harm [23]. A total of 153 young LGBTQ+ people were randomized into the Purrble intervention (Purrble+safety planning; n=77) or treatment-as-usual (TAU, safety planning only; n=76) on a 1:1 basis, stratified by gender identity (cisgender vs transgender and gender diverse [TGD]). Across the 13-week trial, participants in the Purrble condition reported significantly lower ER difficulties, anxiety, and depression symptoms compared to TAU controls. However, analysis indicated a significant interaction for ER and anxiety, whereby these effects only remained significant for cisgender participants and not TGD youth. No effect was observed for self-harm outcomes.

A qualitative process evaluation was conducted at the end of the trial, inviting participants to take part in semistructured interviews to discuss their perspectives and experiences of the trial and Purrble. Process evaluations are crucial elements of RCTs [25], helping researchers to understand how participants perceive and experience the intervention, explaining how interventions may work, its potential integration into daily life, and the trial design itself [26,27]. The in-depth qualitative findings from such methods are particularly important to understand the impact of novel, emerging interventions, such as Purrble, and considering future implementation [28]. Here, the process evaluation, which has not previously been reported on, helps contextualize and offer insight into the statistical findings of the RCT.

This paper reports findings from the qualitative process evaluation conducted following participation in the related RCT [29], exploring the participants’ views and experiences of the Purrble intervention, considering participant engagement and appropriation of Purrble, and beliefs as to whether Purrble supported their ER, mental health, or self-harmful thoughts.

## Methods

### Study Design

We conducted a qualitative process evaluation, comprising semistructured interviews with participants following completion of the 13-week RCT [29]. The process evaluation was embedded within the trial and examined participants’ experiences of the Purrble intervention, with analyses conducted independently and before the quantitative RCT findings were shared with the research team, to aid interpretation of the eventual quantitative results [26].

### Ethical Considerations

Participants gave informed, written consent at enrollment to the RCT. This encompassed consent to be contacted for interview, be interviewed using audio recording, and for transcription processes. The consent form explained how data would be managed, including that only the King’s College London research team would have access to audio recordings, which were stored in encrypted files. Transcription services were required to sign a confidentiality agreement before receiving any audio data, and all identifiable information was removed during transcription. All instances of audio recordings were deleted once transcription was complete. Participants were required to consent to all of these elements in order to take part in the study.

As compensation, all participants were offered £10 (US $13.27) for their time to participate in the interview. As part of the wider RCT, participants kept their Purrbles after the project was completed.

All study procedures were approved by the ethics committee at King’s College London, UK (RESCM-22/23‐34570) and the trial procedures were prospectively registered ahead of participant enrollment (ClinicalTrials.gov NCT06025942) and published [30].

### Patient and Public Involvement

Members of Sprouting Minds, a youth advisory group with lived experience of mental health difficulties and active involvement in the UK Research and Innovation (UKRI) funded Digital Youth program, played a key role in developing the associated RCT and process evaluation, building on their prior contributions to related projects [22,29]. Advisors reviewed and refined the interview schedule preethical submission, ensuring alignment with study priorities and streamlining inquiry to participant experiences of using Purrble. After data collection, advisors were engaged in reviewing the analysis to confirm that developing codes and themes reflected participants’ perspectives, from their interpretations.

### Interventions

As part of the RCT [29], participants were stratified and randomized to either TAU, receiving only the safety planning intervention, or the Purrble condition, where they received safety planning and the Purrble device. Following the final trial survey, all TAU participants also received a Purrble to keep.

#### Safety Planning

All interested participants attended a compulsory safety briefing to enroll within the trial; this ensured that the young people were fully aware of all research and safeguarding procedures to which they were consenting as part of the trial. During this briefing, participants in both conditions cocreated a safety plan with an Applied Suicide Intervention Skills Training (ASIST)-trained researcher, following the Brown and Stanley [31] framework.

Safety plans are a brief collaborative intervention that can help to prevent suicidal behavior [32,33]. The aim is to cocreate an individualized crisis management tool with the participant, such that during a moment of distress they have a physical reminder of their self-harm triggers, potential coping techniques or supports that are known to be helpful, and direct contact information for selected services [34]. Safety plans have been included as standard self-harm safeguarding protocols within previous LGBTQ+ youth research [22,35,36].

#### Purrble Intervention

Purrble is an interactive plush toy-robot (Figure 1), which was developed through co-design with children to support in-the-moment ER [20,21]. Through embedded electronics, vibrations are used to simulate heartbeat patterns: (1) frantic and anxious; (2) slow and relaxed. When turned upside down, or having its ears touched, the robot emits the frantic heartbeat which can be slowed to the relaxed pattern by stroking movements or hugs registered by sensors within the device. Once the robot has been “soothed” for a length of time, it will emit purring or snoring noises to indicate contentment. The transition can be achieved in under 60 seconds but is dependent on the human-device interaction. Further details on the logic model underlying Purrble can be found in Multimedia Appendix 1.

**Figure 1.**
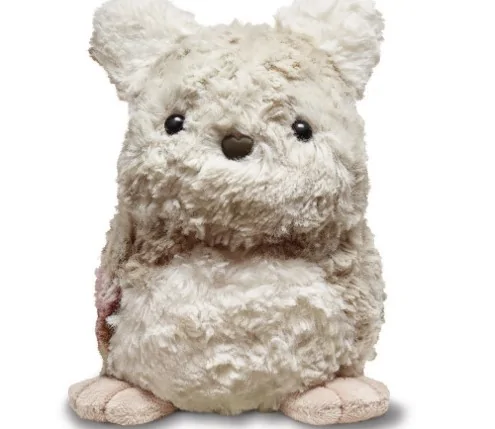
Purrble intervention.

### Recruitment

Once participants had finished their final RCT assessment and TAU participants had been sent their Purrbles, all participants were emailed information about the interview with a link to fill in their availability if they wished to take part. The original aim was to collect data from up to ~40% of the Purrble intervention (n=37) and ~20% of the TAU condition (n=18) [30]. This sample would provide a broad, unselected sample maximizing potential experiences captured, and representing a significant proportion of the trial participants [36].

All interested participants were invited to interview (n=55), with priority given to those in the intervention group to ensure control condition members would have received Purrble and had time to engage with the device before they took part in the interview. Eleven participants did not respond when they were contacted for an interview, while 4 were unable to find a suitable time.

Forty participants (21 intervention; 19 TAU) took part in online, semistructured interviews between August-September 2024. Interview participants were all aged between 16‐25 years, representing a range of sexual orientations and gender identities. In the RCT, randomization was stratified by gender identity with consideration given to these differences within analysis. Within interview participants, 21 identified as cisgender and 19 were transgender or gender diverse. This demographic characteristic is denoted by either (TGD) or Cisgender# (cisgender), for clarity. Twenty-nine participants disclosed confirmed or suspected neurodiversity (eg, autism, attention-deficit hyperactivity disorder [ADHD], and dyslexia), 10 stated they had none, and 1 participant did not offer any data on this. Further descriptives are not offered to prevent participant identification, given the physical nature of the intervention and combination of demographics available.

### Data Collection

The interviews broadly discussed (1) expectations of the RCT and Purrble; (2) whether their safety plans had been used, and to what degree; (3) engagement and appropriation of Purrble; (4) Purrble’s impact on well-being and mental health, including self-harmful thoughts and behaviors; (5) other coping mechanisms; and (6) potential wider impact for Purrble. The semistructured nature of the interview allowed for reflexivity and flexibility to explore participants’ experiences as they are disclosed [37].

Participants were interviewed by AJW and LP; these were single online interviews with only the researcher and participant present. Both researchers created reflexive diaries to capture their thoughts and observations from the interviews; these were used to support later analysis. The interviews typically lasted 28 minutes (range 12-60 minutes) and were audio recorded. These recordings were transcribed verbatim, with identifying information being removed for confidentiality.

### Data Analysis

Qualitative process evaluation analyses were conducted independently and prior to any quantitative analysis of the RCT data to minimize potential bias and support unbiased interpretation of participants’ experiences [26].

As 90% of the interviews were conducted by AJW, initial analysis began during the data collection and was used to determine that data saturation was achieved before recruitment ceased [38]. This was discussed with LP to ensure similarity across interviews and interviewers. Thematic analysis was used following the steps outlined by Braun and Clarke [39,40], with AJW leading the analysis (100% of transcripts), supported by LP who conducted 10% of the interviews and analyzed 25% of the transcripts.

After data familiarization, both authors (AJW and LP) extensively coded transcripts for topics, experiences, perspectives, and opinions relating to the research questions using line-by-line coding, creating their own initial frameworks. Reflexive diaries were used to document initial impressions of interviews, emerging interpretations, notable participant narratives, and potential assumptions arising during engagement with the data. For example, researchers recorded instances where particular issues appeared especially important to participants, alongside reflections on how their own perspectives may influence interpretation. These reflections were revisited throughout coding and theme development to support ongoing reflexivity and critical examination of analytic decisions. The initial coding frameworks were subsequently compared, with similarities, differences, and areas of overlap discussed in relation to the wider dataset and relevant contextual information. These discussions were used to challenge assumptions, explore alternative interpretations, refine conceptual boundaries, and ensure that themes remained grounded in participants’ accounts. This framework was then reflexively discussed among the team to establish credibility and methodological rigor of the analysis.

## Results

Four major themes were identified relating to the research question: (1) “I wouldn’t be here, if I didn’t have my Purrble”; (2) The Companion; (3) Accessibility of Purrble; and (4) “OK, this isn’t for me.” Each theme is described in further detail with supporting subthemes and quotes, with the overarching thematic framework presented in Table 1 with thematic prevalence. Theme prevalence is offered as insight into the generalizability of themes across participants, but higher or lower prevalence does not indicate the importance of a specific experience to an individual. Across interviews, no participants spoke in depth or in detail about using their safety plans.

**Table 1.** Thematic framework of process evaluation.

Themes and subthemes		Prevalence (%)
Theme 1. “I wouldn’t have been here if I didn’t have my Purrble” (T66)		
1a. A moment with Purrble		77.5
1b. Recognizing thought patterns		32.5
1c. “You reach for the Purrble and then try to bring yourself back” (T4)		32.5
1d. Purrble: the displacer		35.0
Theme 2. The Companion		
2a. Comforting presence		87.5
2b. Sidestepping social demands		37.5
2c. Externalizing emotions		17.5
Theme 3. Accessibility of Purrble	
3a. Keep Purrble close		45.0
3b. Purrble doesn’t come out with me		32.5
Theme 4. “OK, this isn’t for me” (T47)	
4a. Overstimulating and overwhelming		37.5
4b. Unsuitable situations		7.5

### Theme 1: “I wouldn’t be here, if I didn’t have my Purrble” (TGD66)

Among participants, Purrble was found to support in-the-moment grounding, which allowed them to regulate their emotions in periods of distress. Over half of participants described using Purrble to manage distress related to self-harm, with grounding functioning as a cross-cutting mechanism across the self-harm cycle (Subtheme 1a). Specifically, participants reported using Purrble when recognizing preceding thought patterns to self-harm (Subtheme 1b), to manage the self-harmful thoughts themselves (Subtheme 1c), or to prevent acting on the urge to self-harm (Subtheme 1d).

#### Subtheme 1a: A Moment With Purrble

One of the most common uses of Purrble was to support ER through grounding:

*[J]ust every time that I felt myself either like panicking or going through a rough spot, I would get into bed or sit with it and then put it on and I’d focus on my breathing and hold it, and calm it down and it also calmed me down*.

[Cisgender20, intervention]

This was supported by the tactile nature of the Purrble device as participants were able to directly interact with the robot and did not need to rely on cognitive strategies:

*I think it was just quite nice having something to hold and sort of, I think even the vibrations of it were sort of quite calming tactically.*

[Cisgender29, intervention]

*It’s just I feel that it’s just nice having like something in front of you to actually interact with, because I found that with a lot of like grounding techniques that like people would usually recommend, it’s really relying a lot on your imagination. There’s like, if you have something physical, it feels like for me at least, it’s a lot more helpful.*

[TGD15, intervention]

#### Subtheme 1b: Recognizing Thought Patterns

Purrble was described as useful for managing anxiety, low mood, or stress. However, participants also demonstrated awareness that these emotional states often functioned as precursors to self-harm, describing how prolonged periods of distress would typically precede the emergence of self-harm-related thoughts. These participants found that they would engage with Purrble during these times to prevent spiraling into distress:

*[B]y virtue of like using it for purely anxiety, that was also probably stopping me from getting into a space where I would feel like I would need to self-harm or something like that.*

[TGD1, intervention]

*Normally if I was like anxious or like was worked up and overthinking. Like normally when I come home, I overthink a lot of what’s happened during the day. But I was able to kind of let the problem settle and actually think, Oh no, I’m just overthinking that. Like it’s not actually that deep and this is just me spiraling and it helps me kind of like ground myself rather than just letting my thoughts run away*.

[Cisgender5, intervention]

#### Subtheme 1c: “You reach for the Purrble and then try to bring yourself back” (TGD4)

*So I ended up using it when I thought like I wasn’t doing so well and I was having thoughts about maybe hurting myself.*

[TGD12, intervention]

Purrble was described as supporting ER while participants were actively experiencing self-harm–related thoughts. In this context, participants used Purrble during the cognitive phase of distress, when thoughts of self-harm were present but had not yet escalated to action. The intervention was described as a mechanism to either directly manage the thoughts as they were occurring—“*don’t think it [Purrble] took anything away, but I think it just reduced my thoughts about it*” (Cisgender9, intervention)—or strategy to de-escalate self-harm ideation allowing participants to access other cognitive strategies:

*[T]erms of like using Purrble to calm down, I think Purrble sort of facilitated that sort of mind switch away [from self-harmful thoughts]. Where I was able to look at things more positively once I had calmed down. So it was more sort of that Purrble allowed me to actually access those kind of thoughts*.

[C49, TAU]

*And so I feel like it’s just in that moment of crisis is when you reach for the Purrble and then try to bring yourself back, like inch yourself back away from the other end of the scale [...] it’s like if you focus on something else, it kind of pulls you back just enough to where you kind of regain your wits about you, and you can then like move back yourself like consciously step back.*

[TGD4, TAU]

#### Subtheme 1d: Purrble: The Displacer

In contrast to Subtheme 1c, participants described using Purrble at points of acute risk, when urges to self-harm had intensified and they felt close to acting on them. Here, Purrble appeared to function as a behavioral interruption:

*I think the emotions I was feeling would normally lead to acts of self-harm, I think Purrble gave me the ability to not just go straight for that.*

[Cisgender29, intervention]

*...more helpful on the occasions where like I really felt like I was about to do something harmful and then I’d be like hang on instead of picking up something that can cause harm, pick up a little thing instead and that was kind of where I find Purrble was most use…*

[Cisgender65, intervention]

Some participants described that Purrble displaced self-harm behaviors, simply as their hands were occupied, therefore meaning they could not access the means to harm themselves:

*I used it for that and it was quite helpful because it just gave me something to do with my hands instead of injuring myself until the urge passed.*

[TGD18, intervention]

The tactile nature of the device also helped participants to avoid hurting themselves, due to outcomes which could impact Purrble:

*You don’t want to hurt yourself when you’re holding it, because then it would stain the Purrble.*

[TGD55, intervention]

### Theme 2: The Companion

Purrble was positioned by participants as a relational tool that provided emotionally meaningful yet free from the expectations associated with human interaction. Participants described experiences of Purrble as a comforting presence (Subtheme 2a), a means of sidestepping social demands (Subtheme 2b), and a mechanism for externalizing emotions (Subtheme 2c). Among the participants, loneliness and isolation associated with LGBTQ+ identity or neurodiversity was discussed at length, with youth suggesting that Purrble had the potential to fill a void for some and thereby encourage repeated engagement. This perception was related to Purrble being a constant presence who was undemanding of external needs.

#### Subtheme 2a: Comforting Presence

Most participants found Purrble comforting in some way; this allowed them to make a connection with the device and repeatedly engage with it when they were dysregulated:

*I really liked using Purrble, yeah that was very relaxing and comforting*.

[Cisgender52, intervention]

*And I think having like a physical item of comfort then is really useful because it sort of grounds you, ties you back into the world that’s you’re not constantly trying to navigate with this huge level of stress that other people don’t seem to have.*

[Cisgender49, TAU]

Purrble was often compared to a stuffed toy or animal, eliciting the same comfort that might come from these. In these accounts, participants did not need Purrble to be doing anything but by simply having it with them, making soothing noises, was calming:

*It was quite nice to have something slow to focus on. And like I’ve always found purring comforting, like I’m a cat person. So it was quite nice to feel like I had a pet with me just sitting with me.*

[TGD49, TAU]

Some participants related this comforting presence as Purrble having a consciousness, which reminded them that they were not alone and supported them to regulate their emotions; *“So it would help me like just see that this little creature has a heartbeat. It’s gonna be OK. We’re alive. And that’s enough. You being alive is enough, you know what I mean? It’s precious.”*

[Cisgender38, TAU]

#### Subtheme 2b: Sidestepping Social Demands

Several participants described Purrble as a friend or companion who did not need anything from them. This allowed them to feel less alone or isolated without having to expend any social energy, which was particularly helpful for those who identified as neurodiverse. This is highlighted by the quote below from TGD1, who is autistic:

*I think as well it’s often difficult to connect with people or sometimes it is. But like often you want comfort. But being with a person who’s got like a whole other mind and sense of self, and stuff like that they’ve got going on and stuff that they need. I’m not saying it’s disparaging, but sometimes you just you need to be self-focused and having something that doesn’t need anything from you, excuse me, and can just be there to mimic that physical comfort from another being. But there’s no social pressure or anything like that.*

[TGD1, intervention]

Participants also felt that Purrble was a constant and reliable connection, which they could not always guarantee from other people:

*... it’s always there and it will never actually like betray you in a sense or out you or anything else like that.*

[TGD66, intervention]

This meant that they did not have to consider social strategies or skills when trying to engage with Purrble:

... *that’s [Purrble] like something else that isn’t going to like ask questions or it’s just going to support you unconditionally, which is quite nice*.

[TGD67, intervention]

#### Subtheme 2c: Externalizing Emotions

Speaking to Purrble was used as a way to externalize and manage emotions:

*I did find myself just kind of like speaking to it sometimes, kind of just venting and saying stuff and it felt a bit better than just saying it to myself.*

[Cisgender20, intervention]

Participants often displayed a lack of self-compassion but were extremely aware of others’ emotional states. Therefore, some opted to externalize their emotions to Purrble and speak to Purrble as a “friend,” which allowed themselves to be compassionate to their own needs in that moment:

*I think when I’m anxious, it’s more like, a kind of, the sort of flipping the narrative as if like Purrble was anxious, as it were me, I would kind of talk to it and that might sound a bit weird, but like I’m soothing it, I will say things out loud that I might need to hear*.

[TGD34, TAU]

This mimicked therapeutic strategies where participants are encouraged to imagine speaking to a child version of themselves:

*So it would be like I mentioned my inner parent taking care of my inner child while I was like comforting Purrble. It would, I’d sort of find it helpful to just say out loud like the things that I would be saying to my inner child and because I’m taking care of a small soft like thing, it’s sort of that I could picture that as my inner child, kind of because it is distressed and like I am distressed. So it’s as I’m calming that down, I’m able to merge those two if that make sense*.

[TGD23, TAU]

### Theme 3: Accessibility of Purrble

Purrble was designed to support in-situ ER; participants found that if they were able to form a habit with the device, it was consistently useful. However, participants needed to have Purrble close to hand when they were distressed to maintain engagement (Subtheme 3a), with some participants describing it as not accessible enough (Subtheme 3b). This limited how useful Purrble could be in moments of dysregulation.

#### Subtheme 3a: Keep Purrble Close

Purrble was described as being kept in a location where participants typically were when they were distressed, for example, in bed or somewhere in the bedroom. It was useful that they could reach over to Purrble and touch it in that moment; this could be to support their grounding, as a soothing and comforting mechanism, or as a distraction:

*I’ve kept it in my bed, and then I’d come back from work every day and then being like instead of like, you know, like hurting myself or punishing myself, I could just get Purrble cause it was right there.*

[TGD67, intervention]

*And so when I was thinking about that [self-harm], it would usually be in times of depression when I can’t feel, but I can’t get out of bed. So just being able to reach over and get it, that can be a solution instead. And yeah, it was really useful and it would kind of be, I’d think about the thoughts and I’m a bit of an overthinker, I would think the thoughts and think about what I wanted to do about them and then I’d think “oh Purrble” and then I’d go for it.*

[Cisgender20, intervention]

A key element of this proximity of use was that Purrble was visible to them; often participants would describe how if Purrble was not close by to them or could not be easily seen, they would forget it was there:

*I think again it needs to be a visible thing because I won’t think about reaching for it if it’s not there.*

[Cisgender27, TAU]

For some participants, this was related to Purrble being a newer practice and therefore was not necessarily in their first thoughts.

#### Subtheme 3b: Purrble Doesn’t Come Out With Me.

Some participants did not feel comfortable taking Purrble outside their homes, limiting its accessibility and utility:

*I didn’t engage with it that often. Cause I found that it was often a little bit out of my way, especially if I was in like, a moment where I was having, like thoughts of self-harm and stuff. […] a lot of the time when I have those thoughts I’d be out and about. I wouldn’t necessarily be home and I didn’t carry this around with me the whole time.*

[Cisgender65, intervention]

This was sometimes related to practical reasons*—“Obviously I couldn’t use her in exams because of the noises”* (TGD66, intervention)—or participants wanting to keep Purrble safe and clean. But more often it was associated with wanting to keep their engagement with Purrble private, feelings of embarrassment, or feeling that Purrble would signal their distress to others:

*I guess one thing would be like if I’m around people, I wouldn’t want to use it because it’s like a signal that I’m feeling distressed, even though, like, I’m not like showing it.*

[TGD23, TAU]

*I was wishing that I could take Purrble to work just for like stresses and annoyances at work but I felt like I didn’t really want people to ask what I was holding.*

[Cisgender7, intervention]

### Theme 4: “OK, this isn’t for me” (T47)

Some participants found that Purrble was not helpful for them, as it was not suited to their needs, coping strategies, or in a few instances their own acknowledged biases: *“I don’t maybe have the patience to have like sat with it and stuff.”* (T47, TAU); *“just didn’t feel right because I’m a 22-year-old male with a cuddly toy.”* (TGD35, intervention). But mainly, this was related to Purrble being unhelpfully stimulating (Subtheme 4a), which made it unsuitable for those with certain experiences or sensitivities (Subtheme 4b).

#### Subtheme 4a: Overstimulating and Overwhelming

For some, there was a cost-benefit balance of engaging with Purrble. It was recognized as helpful to a degree or at some points in the vibration pattern; however, the rapid heartbeat and noises could be off-putting:

*I think for people that are overstimulated it wouldn’t be helpful because I think it is quite stimulating in a sensory way, like you can hear the vibrations, you can feel them.*

[Cisgender34, TAU]

This was irrespective of neurodiversity, where participants specifically spoke about the tactile nature being a benefit for those who identified as neurodiverse.

*I just get slightly mad at Purrble but just keep going. I think it did eventually then have that calming effect where it would still calm down. I would then find my own breathing evening out. But it would just be an extra slight frustration to get there.*

[Cisgender49, TAU]

This overstimulation could also come from participants recognizing that Purrble was stressed and feeling overly empathetically attached to the device. This could occur when they wanted to go through the calming cycle again with Purrble and were concerned about upsetting the device, or when they were at heightened levels of stress and having this mirrored in Purrble was overwhelming:

*[Y]eah I think it was kind of stressing me out a little bit more, I think because I couldn’t calm it down. So then I started feeling like guilty.*

[TGD3, TAU]

Alternatively, when participants had used Purrble to regulate their emotions and had finished engaging with it, accidental movement causing Purrble to become “stressed” again was found to be irritating:

*I was using it to get to sleep again, but it was like you’d nudge it a little bit after you’d quieted it, you had to put a lot of effort in to quiet it down*.

[TGD67, intervention]

#### Subtheme 4b: Unsuitable Situations

Two situations were disclosed where Purrble was thought not to be a suitable option among participants. The first was when 2 participants were experiencing gender dysphoria; this was associated with Purrble not being able to fix those very identity-focused thoughts or that the vibrations were uncomfortably stimulating, reminding one of their own body and how this does not match their gender identity:

*The only time it didn’t really help was when it was feelings directly correlated to dysphoria, that sort of thing. Because well, it’s a lot more complicated.*

[TGD56, intervention]

*Yeah, I did try to use Purrble a lot. […] but the only reason that I struggle to use Purrble was because of the way the Purrble vibrates. Like holding it in your hands kind of works but it doesn’t do much. You gotta hold it against your body, but because I’m like trans as well as like LGBT in the sense of romance type, that holding it against my body triggered dysphoria.*

[TGD55, intervention]

Second, a participant was mindful that holding Purrble while it vibrated could be triggering for those who had suffered from sexual abuse. Participant C57 alluded to their experiences of these, but despite these reservations, participant Cisgender57 was a very active user of Purrble.

*[T]aking that in mind with people that have had problems with sexual abuse and things in the past, it could maybe be an issue for some people. It’s something that it kind of spiked in my mind the first few times I was using the Purrble and that did kind of go away for me. But it was just kind of a little thing that I thought I don’t know whether that for some people, that may be a bit triggering within itself.*

[Cisgender57, TAU]

## Discussion

### Principal Results

The purpose of this qualitative process evaluation was to understand how LGBTQ+ young people perceived and experienced Purrble (delivered alongside safety planning) as a novel intervention to support in-situ ER. Although analyses were conducted independently and prior to access to quantitative outcomes, the findings are highly complementary to those of the main RCT [29]. Specifically, the trial demonstrated that participants in the Purrble intervention condition reported significant reductions in ER difficulties, anxiety, and depressive symptoms compared to TAU controls, which aligns with the present qualitative findings, where participants described in detail the ER strategies they developed and deployed through interacting with Purrble to manage distress.

While the main RCT did not demonstrate statistically significant effects on self-harm outcomes [29], it is important to note that the trial was not powered for self-harm as a primary outcome. These qualitative findings should not be interpreted as evidence that Purrble prevents self-harm or as an explanation for the RCT’s null self-harm findings. Instead, participants described experiences of using Purrble to identify, manage, and respond to distress, which was often related to their self-harm, providing insights into how they perceived Purrble to support their emotional experiences in real time. Some participants felt that engaging with Purrble helped them recognize and regulate distress before it escalated, aligning with previous findings [22]. In this way, the qualitative findings complement the trial results by generating contextual hypotheses regarding how improvements in proximal outcomes (eg, emotion regulation, anxiety, and depression) may be experienced by participants and how such changes might relate to self-harm risk. These proposed mechanisms remain speculative and should be examined directly in future research.

Furthermore, these qualitative findings also help to theorize why differential effects were observed within the RCT [29]. A small number of participants reported that Purrble could trigger gender dysphoria or felt misaligned with identity-related stereotypes. This provides a potential explanatory mechanism for the observed interaction effects in the RCT and may help to account for why Purrble appeared less supportive for some transgender and gender-diverse participants [29] and highlights the importance of considering identity when interpreting engagement with interventions [6].

A key aspect of these findings was participants’ use of Purrble to manage feelings of loneliness and social isolation, both of which were described as contributing to emotional distress. This is particularly noteworthy given evidence that LGBTQ+ individuals experience high levels of loneliness, social rejection, marginalization, and isolation [5,41-44]. Such experiences are often understood within the minority stress framework [45,46], which proposes that sexual orientation and gender identity minority individuals face additional chronic stressors related to their identity, including stigma, discrimination, and expectations of rejection. These experiences can further harm social connectedness and contribute to poorer mental health outcomes. Whereas strong social relationships and supportive networks are consistently associated with reduced psychological distress and lower risk of self-harm among LGBTQ+ populations [5,46,47]. Therefore, participants’ descriptions of Purrble as companion-like suggest that its benefits may extend beyond emotion regulation alone. For some young people, particularly those with limited social support, those experiencing social withdrawal, or those who are neurodivergent and find interpersonal interactions more demanding, Purrble may provide a temporary source of comfort, connection, and perceived support during periods of distress. While not a substitute for human relationships, participants’ accounts suggest that Purrble may help bridge moments of isolation and provide emotional support, particularly when a young person is already feeling overwhelmed or socially tired.

This study highlights the value of incorporating process evaluations within trials. Process evaluations are increasingly recognized as essential for understanding how complex interventions operate and which components drive observed effects [25-27]. However, few RCTs in LGBTQ+ mental health research have incorporated this methodological approach [14,48-51]. In this study, the process evaluation enabled examination of the relative contributions of Purrble and safety planning within the intervention package. Across conditions, participants rarely referenced their safety plans, and when prompted, many struggled to recall them. Instead, narratives consistently centered on Purrble, suggesting that the observed impacts were largely attributable to the device itself rather than the combined intervention (Purrble+safety planning). It is possible that participants’ limited recall or use of their safety plans reflected a lack of need, as few participants described experiencing the type of suicidal crisis for which the Brown and Stanley safety plan was originally designed [31]. Nevertheless, these findings contribute to ongoing debates regarding the practical utility and implementation of safety plans in practice. While safety planning remains a standard component of interventions for individuals at risk of self-harm or suicide in both research contexts [22,32,33,35,52] and UK clinical practice (eg, Improving Access to Psychological Therapies [IAPT]) [34], previous evidence indicates that engagement is often limited during periods of acute distress [53]. Factors such as ambivalence, reduced confidence in usefulness, or forgetting the plan altogether may contribute to this disengagement [54]. The limited prominence of safety plans within participant accounts in this study aligns with and extends these concerns.

One consideration relates to the characteristics of the process evaluation sample. Participants were recruited from those who had taken part in the related trial [29] and consented at enrollment to being contacted for a qualitative interview. While this approach enabled the capture of experiences from individuals with direct exposure to the intervention and minimized participant burden, it may also have introduced self-selection bias. Participants who agreed to be interviewed may have been more engaged with the intervention, more willing to discuss their emotional experiences, or more motivated to share particularly positive or negative views than those who declined participation. In addition, individuals who may have become interested in contributing their perspectives at a later stage were not captured through this recruitment approach. As reasons for declining participation were not collected, it was not possible to determine whether systematic differences existed between interview participants and the wider trial cohort. This should be addressed in future trials investigating the impact of Purrble.

Neurodiversity was also highly represented within the sample and may have influenced participants’ engagement with Purrble. Many participants described the device’s tactile and sensory features as supporting grounding and ER during periods of distress, consistent with previous work highlighting sensory engagement as a key contributor to Purrble’s acceptability [22,23]. However, sensory features were not universally experienced as beneficial, with some participants reporting that the vibrations could be overstimulating and contribute to disengagement. Given that sensory preferences and sensory regulation are important considerations for many neurodivergent individuals, these factors may have shaped both positive and negative experiences of the device [55]. As neurodivergence was not examined systematically within the analysis, it was not possible to determine whether engagement or perceived benefits differed between neurodivergent and neurotypical participants. Consequently, although these findings provide valuable insight into how LGBTQ+ young people may engage with and benefit from Purrble, they are not intended to be representative of all trial participants, but rather to offer potentially transferable insights for similar populations and contexts. From a clinical and policy perspective, these findings have important implications. Demand for psychological support among young people in the UK is substantial: approximately 1 in 5 meet criteria for a probable mental disorder, most commonly anxiety and depression, and anxiety is the leading reason for referral to National Health Service (NHS) child and adolescent mental health services [56,57]. In this context, the cost of providing a Purrble device is relatively low (up to £50 plus £6 shipping [US $66.34+$7.96]) compared with the estimated cost of 1 hour of NHS clinical psychologist time (approximately £69–£75 [$91.55-$99.51]) [58], highlighting its potential as a scalable, low-intensity support option. Purrble may therefore represent a cost-effective intervention, particularly for LGBTQ+ young people who face persistent structural and interpersonal barriers to accessing formal mental health support [9,10,12], although this remains to be established through formal economic evaluation. As an independent, self-directed tool, Purrble can provide support at moments when young people might otherwise disengage from services. Participants’ narratives suggest that it functions as a source of perceived social support, a well-established protective factor against self-harm [59,60], by offering a consistent, nonjudgmental presence that promotes ER strategies.

Crucially, Purrble’s mechanisms of change are transdiagnostic [20], targeting ER processes that underpin diverse mental health and well-being outcomes. This process-level focus, rather than a diagnosis-bound approach, means that Purrble may be suitable across clinical services, educational environments, and medical settings. For example, Purrble may be particularly valuable for young people on waiting lists for mental health services, by promoting foundational ER skills and scaffolding engagement with later therapy. This is a particularly important application context because young people awaiting services are at elevated risk of suicide attempt [61] and frequently report feelings of disengagement and disenchantment prior to treatment [62], whereas the provision of waiting list interventions may help to mitigate these risks [63]. More broadly, socially assistive robots have been shown to be feasible and acceptable within educational and healthcare settings, supporting learning, confidence, and reductions in anxiety [64-66]. Purrble may therefore offer a means of extending such benefits to older adolescents and young adults, including in emotionally demanding situations such as difficult conversations with education staff or managing medical anxiety. Across these settings, the provision of a Purrble device for private, self-directed use represents a relatively low-cost approach with the potential to reduce acute distress.

Our findings provide several directions for additional research. First, studies should examine potential differences in Purrble use and perceived impact across sexual orientations and gender identities, with particular attention to whether specific design features may inadvertently evoke discomfort for young people experiencing gender dysphoria. Such work would inform refinements to ensure the intervention remains affirming and psychologically safe. Where appropriate, future research should also adopt coproduction approaches to ensure that interventions are developed in partnership with those who may use them [67], in this case TGD young people. Indeed, it is unlikely that a single intervention will be effective or acceptable for all, particularly in the context of mental health and self-harm. Individuals differ in their needs, preferences, and circumstances, and interventions should reflect this diversity. The aim, therefore, should be to develop and evaluate a range of evidence-based approaches that can be tailored to different needs and contexts. Such flexibility is likely to maximize engagement, acceptability, and effectiveness across diverse groups. Second, participants often forgot about the safety plan component of the intervention, a common challenge reported in the safety planning literature [53]. Future intervention designs may benefit from incorporating multiple opportunities to revisit and review safety plans in order to enhance engagement and encourage their use during periods of distress. These intervention nudges may promote active interaction with safety plans, requiring individuals to review, update, and personalize content over time rather than passively receiving information. Active choice engagement is typically associated with greater behavioral impact than information provision alone [68]. Third, many participants highlighted neurodiversity, noting that the tactile and sensory aspects of Purrble were especially beneficial during periods of distress. Given the overlap between neurodiverse and LGBTQ+ identities, with evidence indicating that LGBTQ+ youth are approximately twice as likely to be neurodiverse [69,70], that neurodiverse young people are also underserved [71], and the substantial representation of neurodiverse participants within the present study, future research should systematically investigate whether neurodiversity influences uptake, engagement, or perceived benefit of Purrble. This could help disentangle whether the benefits observed are associated with LGBTQ+ experiences specifically, neurodiversity, or the intersection of both identities. Such research may clarify whether participant labels alone mask important individual differences. Finally, findings from the RCT indicate that Purrble improves ER and broader clinical outcomes over a 13-week deployment [29], while qualitative data further suggest young people can use Purrble in order to respond to and manage their distress associated with self-harm. A fully powered trial specifically designed to examine the impact of Purrble on self-harm outcomes is therefore warranted.

### Limitations

Several limitations should also be considered. The findings may be subject to self-selection bias; as although some participants articulated ambivalence toward Purrble and identified contexts in which it was less suitable, it is likely that those who agreed to be interviewed were more positively disposed toward the intervention than the wider trial cohort. Furthermore, as information regarding why RCT participants declined interview participation was not collected, it is not possible to assess whether interview participants differed from nonparticipants. Assessment of those who took part in these process evaluation interviews and those who did not has not been conducted. In addition, while the trial recruited participants with a broad range of sexual orientations and recruitment to interviews aimed to balance gender identity broadly (cisgender vs transgender or gender diverse), sexual orientation was not explicitly incorporated into interview sampling or analytic decisions. Consequently, potential differences in how sexual orientation may have shaped perceptions of Purrble’s utility, appropriation, or engagement were not examined in depth.

### Conclusions

This qualitative process evaluation provides in-depth insight into how LGBTQ+ young people engaged with and interpreted the Purrble intervention following participation in an RCT [29]. Across 4 themes, participants described varied but meaningful ways in which Purrble was appropriated to support in-the-moment emotion regulation, manage distress, and navigate difficult emotional states. Importantly, these findings help to contextualize and theorize why significant improvements were observed in the trial in emotion regulation, anxiety, and depressive symptoms [29]. Participants’ accounts illustrate how Purrble facilitated the development and deployment of practical ER strategies in real-world settings, offering a plausible mechanism through which these outcomes were achieved. The qualitative findings also provide insight into how participants understood the relationship between Purrble, emotional distress, and self-harm-related experiences, offering perceptions of how Purrble might support the management of distress and self-harm urges as described in participants’ individual narratives. However, the findings should not be used to explain the absence of statistically significant effects on self-harm outcomes observed in the RCT, given the subjective nature of individual narratives and qualitative analysis. Finally, the variability captured across themes underscores that Purrble is not universally acceptable or beneficial, and that individual differences, such as gender identity, preferences, or past experiences, can shape engagement with the intervention, suggesting further research is needed to understand the value of Purrble with such populations. Overall, this study highlights the potential of low-intensity, accessible interventions like Purrble to support ER among LGBTQ+ young people, while also emphasizing the importance of understanding for whom and under what conditions such interventions are most effective.

## Supplementary material

10.2196/103590 Multimedia Appendix 1 Logic model of Purrble.

## Abbreviations

ADHD: attention-deficit hyperactivity disorder
ASIST: Applied Suicide Intervention Skills Training
ER: emotion regulation
IAPT: improving access to psychological therapies
LGBTQ+: Lesbian, gay, bisexual, transgender, queer, and questioning
NHS: National Health Service
RCT: randomized controlled trial
TAU: treatment-as-usual
TGD: transgender and gender diverse
UKRI: UK Research and Innovation

## References

[1] Marchi M, Arcolin E, Fiore G (2022). Self-harm and suicidality among LGBTIQ people: a systematic review and meta-analysis. Int Rev Psychiatry.

[2] Jadva V, Guasp A, Bradlow JH, Bower-Brown S, Foley S (2023). Predictors of self-harm and suicide in LGBT youth: The role of gender, socio-economic status, bullying and school experience. J Public Health (Oxf).

[3] Hughes E, Rawlings V, McDermott E (2018). Mental health staff perceptions and practice regarding self-harm, suicidality and help-seeking in LGBTQ youth: findings from a cross-sectional survey in the UK. Issues Ment Health Nurs.

[4] Liu RT, Walsh RFL, Sheehan AE, Cheek SM, Carter SM (2020). Suicidal ideation and behavior among sexual minority and heterosexual youth: 1995-2017. Pediatrics.

[5] Liu RT, Mustanski B (2012). Suicidal ideation and self-harm in lesbian, gay, bisexual, and transgender youth. Am J Prev Med.

[6] Fish JN (2020). Future directions in understanding and addressing mental health among LGBTQ youth. J Clin Child Adolesc Psychol.

[7] (2025). Suicide worldwide in 2021: global health estimates. World Health Organization.

[8] di Giacomo E, Krausz M, Colmegna F, Aspesi F, Clerici M (2018). Estimating the risk of attempted suicide among sexual minority youths: a systematic review and meta-analysis. JAMA Pediatr.

[9] McIntyre J, Daley A, Rutherford K, Ross LE (2011). Systems-level barriers in accessing supportive mental health services for sexual and gender minorities: insights from the provider’s perspective. Can J Community Ment Health.

[10] (2018). LGBT in Britain: health report. Stonewall.

[11] Williams AJ, Nielsen E, Coulson NS (2020). “They aren’t all like that”: perceptions of clinical services, as told by self-harm online communities. J Health Psychol.

[12] Whaibeh E, Mahmoud H, Vogt EL (2020). Reducing the treatment gap for LGBT mental health needs: the potential of telepsychiatry. J Behav Health Serv Res.

[13] Liu Y, Wu YC, Fu H, Guo WY, Wang X (2023). Digital intervention in improving the outcomes of mental health among LGBTQ+ youth: a systematic review. Front Psychol.

[14] Gilbey D, Morgan H, Lin A, Perry Y (2020). Effectiveness, acceptability, and feasibility of digital health interventions for LGBTIQ+ young people: systematic review. J Med Internet Res.

[15] Rauschenberg C, Schick A, Goetzl C (2021). Social isolation, mental health, and use of digital interventions in youth during the COVID-19 pandemic: a nationally representative survey. Eur Psychiatry.

[16] Sloan E, Hall K, Moulding R, Bryce S, Mildred H, Staiger PK (2017). Emotion regulation as a transdiagnostic treatment construct across anxiety, depression, substance, eating and borderline personality disorders: a systematic review. Clin Psychol Rev.

[17] Clapham R, Brausch A (2024). Internalizing and externalizing symptoms moderate the relationship between emotion dysregulation and suicide ideation in adolescents. Child Psychiatry Hum Dev.

[18] Wolff JC, Thompson E, Thomas SA (2019). Emotion dysregulation and non-suicidal self-injury: a systematic review and meta-analysis. Eur Psychiatry.

[19] Sheppes G, Suri G, Gross JJ (2015). Emotion regulation and psychopathology. Annu Rev Clin Psychol.

[20] Slovák P, Theofanopoulou N, Cecchet A (2018). I just let him cry…” Designing socio-technical interventions in families to prevent mental health disorders. Proc ACM Hum Comput Interact.

[21] Theofanopoulou N, Isbister K, Edbrooke-Childs J, Slovák P (2019). A smart toy intervention to promote emotion regulation in middle childhood: feasibility study. JMIR Ment Health.

[22] Williams AJ, Townsend E, Naeche N (2023). Investigating the feasibility, acceptability, and appropriation of a socially assistive robot among minority youth at risk of self-harm: results of 2 mixed methods pilot studies. JMIR Form Res.

[23] Williams AJ, Freed M, Theofanopoulou N (2023). Feasibility, perceived impact, and acceptability of a socially assistive robot to support emotion regulation with highly anxious university students: mixed methods open trial. JMIR Ment Health.

[24] Chubinidze D, Li Z, Croft P, Nodding B, Slovak P, Tchanturia K (2025). “Calming the nightmares”: a qualitative study of a socially assistive robot for sensory and emotional support in individuals with eating disorders and PTSD. PLoS ONE.

[25] French C, Pinnock H, Forbes G, Skene I, Taylor SJC (2020). Process evaluation within pragmatic randomised controlled trials: what is it, why is it done, and can we find it?-a systematic review. Trials.

[26] Moore G, Audrey S, Barker M (2014). Process evaluation in complex public health intervention studies: the need for guidance. J Epidemiol Community Health.

[27] Sekhon M, Cartwright M, Francis JJ (2017). Acceptability of healthcare interventions: an overview of reviews and development of a theoretical framework. BMC Health Serv Res.

[28] Diepeveen S, Ling T, Suhrcke M, Roland M, Marteau TM (2013). Public acceptability of government intervention to change health-related behaviours: a systematic review and narrative synthesis. BMC Public Health.

[29] Williams AJ, Rhodes CA, Cleare S (2026). A socially assistive robot to support mental wellbeing in LGBTQ+ young people at risk of self-harm: a randomized controlled trial. Nat Med.

[30] Williams AJ, Cleare S, Borschmann R (2024). Enhancing emotion regulation with an in situ socially assistive robot among LGBTQ+ youth with self-harm ideation: protocol for a randomised controlled trial. BMJ Open.

[31] Stanley B, Brown GK (2012). Safety planning intervention: a brief intervention to mitigate suicide risk. Cogn Behav Pract.

[32] Abbott-Smith S, Ring N, Dougall N, Davey J (2023). Suicide prevention: what does the evidence show for the effectiveness of safety planning for children and young people? - a systematic scoping review. J Psychiatr Ment Health Nurs.

[33] Nuij C, van Ballegooijen W, de Beurs D (2021). Safety planning-type interventions for suicide prevention: meta-analysis. Br J Psychiatry.

[34] (2022). Self-harm: assessment, management and preventing recurrence. National Institute for Health and Care Excellence.

[35] Williams AJ, Arcelus J, Townsend E, Michail M (2023). Understanding the processes underlying self-harm ideation and behaviors within LGBTQ+ young people: a qualitative study. Arch Suicide Res.

[36] Green J, Thorogood N (2018). Qualitative Methods for Health Research.

[37] Petrescu SH, Lazar A, Cioban C, Doroftei I (2017). Qualitative Research in Regional Geography: A Methodological Approach.

[38] Guest G, Namey E, Chen M (2020). A simple method to assess and report thematic saturation in qualitative research. PLoS ONE.

[39] Braun V, Clarke V (2006). Using thematic analysis in psychology. Qual Res Psychol.

[40] Braun V, Clarke V (2019). Reflecting on reflexive thematic analysis. Qualitative Research in Sport, Exercise and Health.

[41] Oeser P, Wellendorff T, Napierala H, Bolster M, Gellert P, Herrmann W (2025). Social networks and loneliness differ between LGBTIA and cis-heterosexual persons: results from a two-wave survey in Germany. Sci Rep.

[42] Elmer EM, van Tilburg T, Fokkema T (2022). Minority stress and loneliness in a global sample of sexual minority adults: the roles of social anxiety, social inhibition, and community involvement. Arch Sex Behav.

[43] Hajek A, König HH, Blessmann M, Grupp K (2023). Loneliness and social isolation among transgender and gender diverse people. Healthcare (Basel).

[44] McDanal R, Schleider JL, Fox KR, Eaton NR (2023). Loneliness in gender-diverse and sexual orientation-diverse adolescents: measurement invariance analyses and between-group comparisons. Assessment.

[45] Meyer IH (2003). Prejudice, social stress, and mental health in lesbian, gay, and bisexual populations: conceptual issues and research evidence. Psychol Bull.

[46] Meyer IH (2015). Resilience in the study of minority stress and health of sexual and gender minorities. Psychol Sex Orientat Gend Divers.

[47] Bird K, Arcelus J, Matsagoura L, O’Shea BA, Townsend E (2024). Risk and protective factors for self-harm thoughts and behaviours in transgender and gender diverse people: a systematic review. Heliyon.

[48] Kirchner S, Till B, Plöderl M, Niederkrotenthaler T (2022). Effects of “It Gets Better” suicide prevention videos on youth identifying as lesbian, gay, bisexual, transgender, queer, or other sexual or gender minorities: a randomized controlled trial. LGBT Health.

[49] Pachankis JE, Harkness A, Maciejewski KR (2022). LGBQ-affirmative cognitive-behavioral therapy for young gay and bisexual men’s mental and sexual health: a three-arm randomized controlled trial. J Consult Clin Psychol.

[50] Pachankis JE, McConocha EM, Reynolds JS (2019). Project ESTEEM protocol: a randomized controlled trial of an LGBTQ-affirmative treatment for young adult sexual minority men’s mental and sexual health. BMC Public Health.

[51] Goldbach JT, Rhoades H, Mamey MR, Senese J, Karys P, Marsiglia FF (2021). Reducing behavioral health symptoms by addressing minority stressors in LGBTQ adolescents: a randomized controlled trial of Proud & Empowered. BMC Public Health.

[52] Williams AJ, Arcelus J, Townsend E, Michail M (2022). Feasibility and acceptability of experience sampling among LGBTQ+ young people with self-harmful thoughts and behaviours. Front Psychiatry.

[53] Czyz EK, King CA, Biermann BJ (2019). Motivational interviewing-enhanced safety planning for adolescents at high suicide risk: a pilot randomized controlled trial. J Clin Child Adolesc Psychol.

[54] Micol VJ, Prouty D, Czyz EK (2022). Enhancing motivation and self-efficacy for safety plan use: Incorporating motivational interviewing strategies in a brief safety planning intervention for adolescents at risk for suicide. Psychotherapy (Chic).

[55] Sabinson E (2024). The pictorial is neurodivergent: a case for more fidgets and fewer fixes.

[56] Children’s Commissioner for England (2024). Children’s mental health services 2023-24. https://www.childrenscommissioner.gov.uk/resource/childrens-mental-health-services-2023-24/.

[57] NHS Digital (2023). Mental health of children and young people in England, 2023. https://digital.nhs.uk/data-and-information/publications/statistical/mental-health-of-children-and-young-people-in-england/2023-wave-4-follow-up.

[58] Jones K, Weatherly H, Birch S (2024). Unit costs of health and social care 2024 manual. Personal Social Services Research Unit (University of Kent).

[59] Snapp SD, Watson RJ, Russell ST, Diaz RM, Ryan C (2015). Social support networks for LGBT young adults: low cost strategies for positive adjustment. Fam Relat.

[60] McConnell EA, Birkett M, Mustanski B (2016). Families matter: social support and mental health trajectories among lesbian, gay, bisexual, and transgender youth. J Adolesc Health.

[61] (2022). 52% increase in young people waiting over a year for mental health support. YoungMinds.

[62] Westin AML, Barksdale CL, Stephan SH (2014). The effect of waiting time on youth engagement to evidence based treatments. Community Ment Health J.

[63] Valentine AZ, Hall SS, Sayal K, Hall CL (2024). Waiting-list interventions for children and young people using child and adolescent mental health services: a systematic review. BMJ Ment Health.

[64] Crossman MK, Kazdin AE, Kitt ER (2018). The influence of a socially assistive robot on mood, anxiety, and arousal in children. Prof Psychol Res Pract.

[65] Kabacińska K, Prescott TJ, Robillard JM (2021). Socially assistive robots as mental health interventions for children: a scoping review. Int J of Soc Robotics.

[66] Silvera-Tawil D, Yates CR Socially-assistive robots to enhance learning for secondary students with intellectual disabilities and autism.

[67] Mendes J de A, Mahamud A, Doherty S (2025). “Co-Production Is Caring”: young people’s reflections on responsible and dialogic co-production in youth mental health. Health Expect.

[68] Mertens S, Herberz M, Hahnel UJJ, Brosch T (2022). The effectiveness of nudging: a meta-analysis of choice architecture interventions across behavioral domains. Proc Natl Acad Sci U S A.

[69] Bränström R, Hughes TL, Pachankis JE, Patterson CJ, D’Augelli AR (2024). Global LGBTQ Health: Research, Policy, Practice, and Pathways.

[70] Lai MC (2025). Editorial: towards an intersectional understanding of sexual identity, mental health, neurodivergence, and beyond. J Am Acad Child Adolesc Psychiatry.

[71] Ashworth E, Saini P, Hanlon C (2026). Over-represented and under-served: autistic children and young people seeking help for suicidal crisis in a UK emergency department. J Autism Dev Disord.

