# Supplementary material for: Purrble Use Among LGBTQ+ Young People With Self-Harm Experiences: Findings From a Qualitative Process Evaluation of a Randomized Controlled Trial
Source: JMIR Form Res. 2026 Oct 27;10:e103590. doi: 10.2196/103590 (PMC13712646; doi:10.2196/103590)
Supplement: Supplementary file 1 [file formative-v10-e103590-s001.docx]

**Supplementary materials: 1**

**Purrble logic model**

Purrble operates on 3 levels:

1. Purrble directly provides in-the-moment soothing support in naturally occurring emotional moments when one would attempt to calm down. The physical and interactive design aims to tap into known regulatory factors using the extended Process Model of Emotion Regulation (Gross, 2015). This focuses on two separate stages of the ER process; i) the attentional deployment stage (Aldao et al., 2015; Sheppes & Gross, 2012; Sheppes et al., 2014; Farb et al., 2014) by shifting attention from the emotional situation towards interacting with the device, and ii) the response modulation stage (Beetz et al., 2012; Dore et al., 2017; Reeck et al., 2016; Coan et al., 2006; Crossman et al., 2018; Rabbit et al., 2015) by facilitating down-regulation through pleasant tactical interactions simulating the emotion regulatory effect of human-animal interactions.

2. Mechanisms of Purrble are designed to facilitate long-term engagement, by building on positive subjective experiences of in-the-moment soothing. As the device is framed as an anxious creature needing to be cared for, the key driver is that interactions are framed as helping regulate others’ emotions (Dore et al., 2017; Cosley et al., 2010; Taylor, 2011), alongside facilitating a sense of relationship and responsibility for the wellbeing of the creature (Turkle, 2007; Hayashi & Kato, 2016; Donath, 2004; Lee et al., 2010).

3. Through repeated, soothing, and positive interactions with Purrble over time, it is anticipated that there will be a shift in ER practices and implicit beliefs about emotions (Ford & Gross, 2018), specifically the controllability of one’s emotions (Schleider & Weisz, 2016).
